# Progress in the Treatment of Empyema

**Published:** 1921-09

**Authors:** J. A. Nixon

**Affiliations:** Physician to the Bristol Royal Infirmary; Consulting Physician to Southmead Infirmary; and formerly Consulting Physician, B.E.F.


### PROGRESS IN THE TREATMENT OF EMPYEMA.

BY

J. A. Nixon, C.M.G., M.D. (Cantab.), F.R.C.P.,

*Physician to the Bristol Royal Infirmary ; Consulting Physician to Southmead Infirmary ; and formerly Consulting Physician, B.E.F.*

---

The responsibility for the treatment of empyema is frequently shared by the physician and the surgeon. The majority of cases come in the first instance under the physician's care, and he subsequently calls upon the surgeon to operate for the relief of the condition. The method of operating has for many years past been more or less stereotyped, and no very general dissatisfaction has been expressed at the results. Powell and Hartley, in the latest edition of their book *Diseases of the Lungs and Pleuræ,* state that "the treatment of suppurative pleurisy does not differ essentially from that of other purulent collections. The pus must be evacuated as speedily and as thoroughly as possible, and its reaccumulation prevented by the provision of free drainage." As to the early and complete evacuation of pus there can be no two opinions, but there is room to question whether the plan usually adopted for providing free and continuous drainage by resecting portions of ribs and inserting rubber drainage tubes is in accordance with the best principles of modern surgery. There was a time when this method of treatment was in vogue for appendicitis, or at least for appendix abscess, but it is now rarely resorted to. Does this method really represent an universally accepted rule of modern surgery?

The advances made in thoracic surgery by Lilienthal, Tuffier, Duval and Gask seem little known, and in hospitals it is not unusual for a physician to tell his house-physician to open an empyema and put in a drainage tube. One wonders what his surgical colleagues would say if he dealt similarly with cases of suppurative appendicitis occurring in his wards.

Apart, however, from the question of whether better methods exist, some responsibility lies upon the physician if he does not point out that the prevailing methods are unsatisfactory, and does not urge the surgeon to seek improved methods. For it may well happen that by silence progress may be hindered because the need for progress is not recognised. Routine resection and drainage undoubtedly save many lives, and not infrequently good functional recovery takes place in the affected lung. But we cannot be ignorant of the numerous instances in which the patient recovers with a sadly impaired lung, with a lung that remains hopelessly contracted, or even with a sinus in the chest wall which obstinately refuses to close. These are failures which in my opinion ought not to be regarded as inevitable. Even if no better procedure were known, these failures should set us thinking how to improve our therapeutic measures.

There appear to be certain points in the treatment of empyema which are accepted almost without question, and I think they stand in the way of progress. For instance :—

Resection and drainage are held to be fundamental principles governing the treatment of empyema.

The object sometimes aimed at in operating is the falling-in of the chest wall on to the unexpanded lung rather than the rapid re-expansion of the lung out to the chest wall.

Washing out of the thoracic cavity is considered dangerous because of " pleural shock."

Collapse of a lung is thought to depend upon air-pressure inside and outside the thorax.

It is believed that after operation expansion of the lung can be obtained by breathing exercises irrespective of the condition of the pleura.

Decortication of the lung is regarded as essentially a late procedure instead of being resorted to whenever possible at the primary operation.

Estlander's operation for thoracoplasty is not yet considered a belated confession of a series of therapeutic failures.

Detailed consideration of these points may lead in the first place to a realisation of the need for better methods, and in the second place may help to suggest the nature of the improvements.

*Resection and Drainage.*—Experience of pleural infections from chest wounds shows that, in a certain proportion of cases if frequent and early aspiration is resorted to, resection can be avoided altogether and permanent collapse of the lung would be less often seen. It became an axiom of battle surgery of the chest that fluid should never be allowed to remain in a chest. It is a very old observation that pneumococcal empyemas sometimes clear up after aspiration only. My impression is that this would prove the case more often if aspiration were performed earlier and with much smaller collections of fluid in pneumonia than is now the customary practice. There is a tendency to delay too long in exploring and aspirating pneumococcal effusions.

But when it is evident that pus continues to collect in spite of aspirations, it is not justifiable to take for granted that resection and evacuation of the pus must be followed by open drainage. I believe a time will come when open drainage of a chest will be regarded as a surgical abomination. It is a procedure to be avoided by every means in our power. If unfortunately it seems necessary to drain a chest temporarily, the aim should be to close it as soon as possible. Under no circumstances should a sucking wound be left. The dangers of a super-imposed infection of the pleural cavity are not widely enough realised. A mixed infection appears to be more dangerous to life, to interfere more with re-expansion of the lung, and to retard convalescence more than the majority of simple infections. This is particularly true of pneumococcal infections. There is great difficulty in avoiding super-infection of a chest with open drainage.

*Falling-in of chest wall or re-expansion of lung.*—There still seems to be an idea that when a chest is opened for empyema it is advantageous to remove considerable portions of one or more ribs in order that the chest wall may fall in upon the contracted lung. The rapidity with which a collapsed lung can re-expand if freed from adhesions does not appear to be known. I had accidental proof of this fact during the war. A patient on whom complete thoracotomy was performed had, by mistake, a radiogram taken of his chest within two hours of his operation. The lung, which had been fully collapsed at the operation, was found both by screening and by a photographic plate to be almost completely re-expanded. The thoracic wound had been hermetically closed and covered with "glue." Evidently air in a pleura cavity is no bar to re-expansion when there are no adhesions and the visceral pleura has been repaired. Whenever possible the pleural cavity should be explored with the hand, flakes of lymph cleared out, and a thorough toilet of the pleura performed.

*Pleural shock.*—It has been thought that the pleura cannot be handled for fear of pleural shock. There may be good reason for not irrigating the cavity, as there is for not irrigating the peritoneal cavity; but the reason is not an excessive sensitiveness of the pleura, the objection is based on the risks of spreading infection to parts which it may not have already reached. Opening the pleural cavity and handling the lung can be done with impunity if care be exercised not to allow displacement of the mediastinum and contents. If the mediastinal contents are gently held in place with tenaculum forceps a patient under local anæsthesia experiences very little discomfort and no shock when the thorax is opened, the cavity explored with the hand, and the lung even "delivered" outside the chest for examination and repair. If, however, the heart is allowed to swing out of position grave shock may occur immediately. I have seen a patient, whose heart was pinned in position by a small fragment of metal which attached the pericardium to the diaphragm, almost die on the operating table when the heart was released and allowed to swing to the opposite side of the chest, although a total pneumothorax had caused him little discomfort either at the time of wounding or after the thorax had been widely opened at the beginning of the operation. It is very striking how very little distress is caused by sudden pneumothorax when the heart is retained in position by old adhesions. I have recently seen an example of this in a soldier who was wounded in 1915 and his pneumothorax dating from that time was only discovered accidentally in 1921. His heart is held in position by adhesions which were probably present before he was wounded. In cases showing extreme distress relief is instantaneous when after opening the chest for thoracotomy the heart is gently drawn back into position.

*Collapse of the lung depending on air in the pleural cavity.*—This is a factor which is greatly over-estimated. If a valve pneumothorax is present constant pumping of air into the cavity may indeed exert sufficient pressure to prevent a lung expanding. But otherwise a lung, as I have mentioned earlier in this paper, can re-expand within two hours of a complete thoracotomy. Experience with artificial pneumothorax for phthisis shows the futility of expecting air to remain unabsorbed when the lung has retained any capacity for expanding. It is on this account that nitrogen is used for replacement rather than air. This makes all mechanical devices for valvular drainage of empyema unnecessary. If the lung is not held down by adhesions, and if air is not being pumped into the pleural cavity through a hole in the lung, or if the lung is not kept contracted by interstitial fibrosis valve drains are not required; if one of these factors is present valve drains will be powerless to effect a re-expansion.

*The value of breathing exercises.*—For the reasons just mentioned breathing exercises are apt to be works of supererogation. To set a patient to blow into bottles unless the lung is unhampered is a waste of the patient's time and energy. We ought first to ascertain that no external hindrances, such as thickening and adhesions of the pleura, exist before expecting breathing exercises to reinflate a collapsed lung. My experience of wounded lungs showed that the toilet of the pleura contributed far more effectively to the re-expansion of the lung than did exercises; we eventually abandoned them altogether, as they only fatigued the patient uselessly. A much smaller experience in the treatment of empyema from disease has convinced me that the same considerations hold good. With a well-cleaned pleural cavity a lung expands satisfactorily without exercises.

*Decortication of the lung.*—It is most important that if decortication is to be performed it should be done early. I mean it should be aimed at when the primary operation is performed. At this stage decortication may be a simple affair; the fibrin may not have organised into the dense covering met with at later stages. The decortication may amount to no more than wiping off caseous material. There may be no tough fibrous coat to be incised and peeled laboriously away. But it may be necessary to disinfect the pleura in some way in order to prevent reaccumulation of lymph. A perfect method has not yet been discovered. Temporary irrigation by the Carrel method is the best plan I have seen. It must not be continued too long, and it must be borne in mind that Dakin's solution applied to the pleura causes the secretion of a curious gelatinous exudate which is very puzzling unless its cause is known. It does no harm and will always stop if the irrigation is discontinued. Complete closure of the chest at the earliest opportunity should be aimed at, and for this purpose the bacteriological state of the pleura must be investigated daily.

*Estlander's operation.*—Thoracoplasty as devised by Estlander seems based on the assumption that since the lung has collapsed the best that can be done is to accept the situation and make the chest wall collapse too. Tuffier has well designated it "Estlander's ill-advised operation." It is time we discovered some better means of restoring the function of the lung rather than by closing the door to any prospect of re-expansion. I believe that if thorough exploration of the pleural cavity were performed either at the primary operation for empyema or as early as possible when it appears likely that the lung is not expanding, this form of thoracotomy, which merely sets a seal upon an embarrassing deformity, would fall into complete and deserved disuse. Even in late cases, where the lung is surrounded by considerable pleural thickening, decortication should always be preferred to thoracoplasty. A lung which has been compressed for years will expand again if the fibrous envelope is either stripped off or freely enough incised.

Complete and early thoracotomy is, however, not to be advocated indiscriminately for all cases of empyema whatever their cause. In exploring new methods of treatment judicious selection of cases must be made. Tuberculous empyemata are unsuitable for operative measures which at present are scarcely out of the experimental stage. Experience has shown that acute streptococcal empyema is best treated by aspiration whilst the patient is acutely ill. The results of early resection were most discouraging during the influenza epidemic of 1918–19; but it was found that if aspiration was performed early, and if necessary repeatedly, many cases cleared up without resection or were tided through until they were fit to stand the operation at a later stage of their illness. The most favourable cases for complete thoracotomy are those due to pneumococcal infection.

In any case the operation should consist of a wide incision allowing good access to the pleural cavity, which should be inspected as thoroughly as possible, explored with the hand, all lymph and fibrin removed, and the chest wall completely closed at the time of operation. Resection of the rib should be avoided, so that when the operation is finished the wall of the thorax may be restored with the least possible permanent damage. Repeated aspiration may be required afterwards. But in any circumstances expansion is likely to be better if the chest wound has been closed, even if it gives way later.

My whole plea is that ultimate restoration of function should be aimed at, as well as immediate saving of life.

